# Multiple organ failure and death caused by *Staphylococcus aureus* hip infection: A case report

**DOI:** 10.1515/biol-2022-0481

**Published:** 2022-09-14

**Authors:** Dechao Cai, Xiao Ma, Yukuan Zhou, Yakun Zhu, Haoran Yu, Wendan Cheng

**Affiliations:** Department of Orthopedics, The Second Hospital of Anhui Medical University, 678 Furong Road, Hefei, 230601, China; Department of Orthopedics, People’s Hospital of Wuhe County, Anhui Province, 123 Huihe Road, Chengguan Town, Wuhe County, Bengbu City, China

**Keywords:** suppurative arthritis, *Staphylococcus aureus* infection, multiple organ failure, case report

## Abstract

Suppurative arthritis has an acute onset and mostly affects old people and children. Recently, the incidence of adult suppurative hip arthritis, as well as its serious consequences, has increased. The deep hip joint and surrounding hypertrophic muscle tissue limit physical examination. Furthermore, they may cause variable and atypical symptoms of suppurative hip arthritis, possibly inducing delayed diagnosis and treatment. This atypical presentation is uncommon, causing delayed diagnosis and treatment, thus worsening the outcomes. We herein report the case of a 58-year-old man with *Staphylococcus aureus* (*S. aureus*) septicemia and multiple organ failure due to left pyogenic arthritis of the hip. The patient’s early symptoms were extremely atypical given that he only presented hip pain. Moreover, there was no obvious history of trauma or inflammatory manifestations, such as fever or local swelling, and laboratory examination results and imaging findings were atypical. However, the disease progressed rapidly, developing into systemic sepsis within a short period of time followed by multiple organ failure and death. Early diagnosis and effective treatment of *S. aureus* hip arthritis are essential to avoid poor outcomes.

## 1 Introduction

*Staphylococcus aureus (S. aureus*) is the most common causative pathogen in patients with infectious arthritis [1,2]. *S. aureus* is a common human pathogen and one of the leading causes of hospital-acquired bacteremia worldwide [3]. Methicillin-resistant *S. aureus* has similar virulence and pathogenicity as other *S. aureus* strains. Septic shock due to *S. aureus* is rare. Metastatic abscesses are characteristic of *S. aureus* infections, with approximately 50% of infected patients experiencing such abscesses during the course of the disease. The most common types of metastatic abscesses are multiple lung infiltrates and lung abscesses, followed by liver abscesses, suppurative meningitis, osteomyelitis, and subcutaneous abscesses. *S. aureus* bacteremia poses a higher risk of developing into severe sepsis than other bacteremias, inducing death and posing a high risk in immunodeficient individuals, those who have undergone major surgery, and old people. Currently, no vaccine against *S. aureus* infection in humans exists [4]. We herein report a case of pyogenic arthritis of the hip caused by *S. aureus* with extremely atypical and early symptoms that rapidly developed and progressed to systemic septicemia causing multiple organ failures and eventually death. This study aimed to address improving vigilance against the causes of pain, initiating early effective intervention, and avoiding serious consequences.

## 2 Case report

A 58-year-old male patient was admitted to the hospital with left hip pain and limited mobility for 1 week. The pain was obvious on admission, and the visual analog scale score, which is a pain score used to evaluate the severity of pain by visual simulation and divided into 10 equal parts using a ruler, with 0 indicating no pain, 1–3 indicating mild pain, 4–6 indicating moderate pain, and 7–10 indicating severe pain, was 5. His medical history revealed chronic obstructive pulmonary disease, hypertension, gout, and urinary stones. Prior to the onset of hip pain, he had a history of heavy physical labor. The patient had no obvious history of trauma or signs of infection elsewhere in the body. His vital signs on admission were as follows: temperature, 37.0°C; pulse, 67 beats/min; respiration, 18 cycles/min; and blood pressure, 118/70 mmHg. Physical examination findings were as follows: good general condition, clear respiratory sounds bilaterally, and normal heart sounds and rhythm with no pathological murmurs. There was no obvious pelvic deformity, and the skin over both hips was intact with no redness, swelling, or rupture. Although not warm, the skin showed deep tenderness at the midpoint of the groin in the left hip (+). The “4” test revealed the left hip (+) and right hip (−). The Thomas sign was positive and negative on the left and right hips, respectively. Other findings were as follows: pelvic separation squeeze test (−); and longitudinal percussion pain in the left lower extremity (+) and right hip (−). The range of motion of the left hip was as follows: flexion, 80°; extension, 0°; external rotation, 20°; internal rotation, 20°; abduction, 10°; and adduction, 10°. The range of motion in the right hip was normal. The motion sensation and blood supply in the affected limbs were good, with no obvious abnormalities.

**Figure 1:**
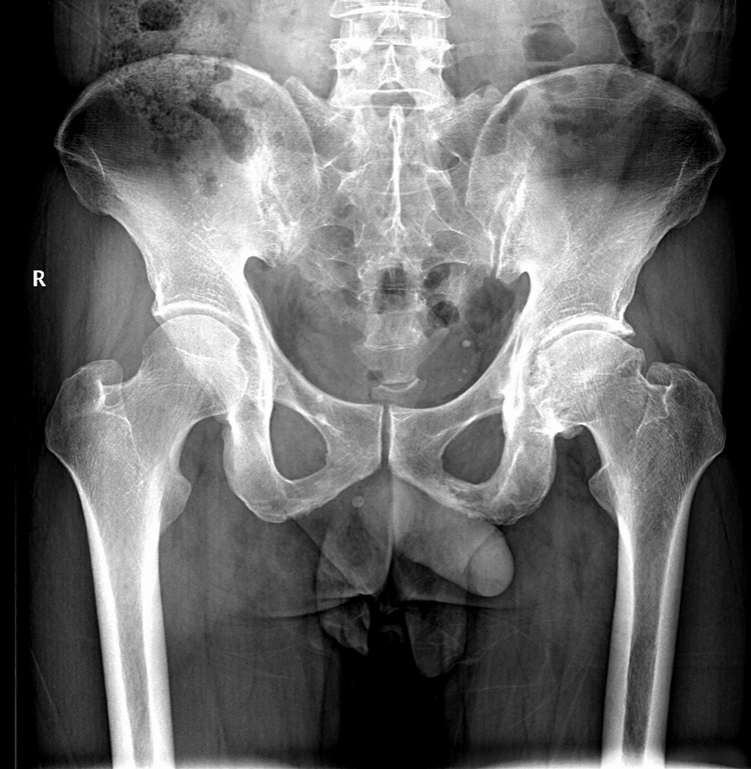
Plain radiograph of the pelvis shows deformation of the left femoral head, narrowing of hip space, and osteophyte hyperplasia.

**Figure 2:**
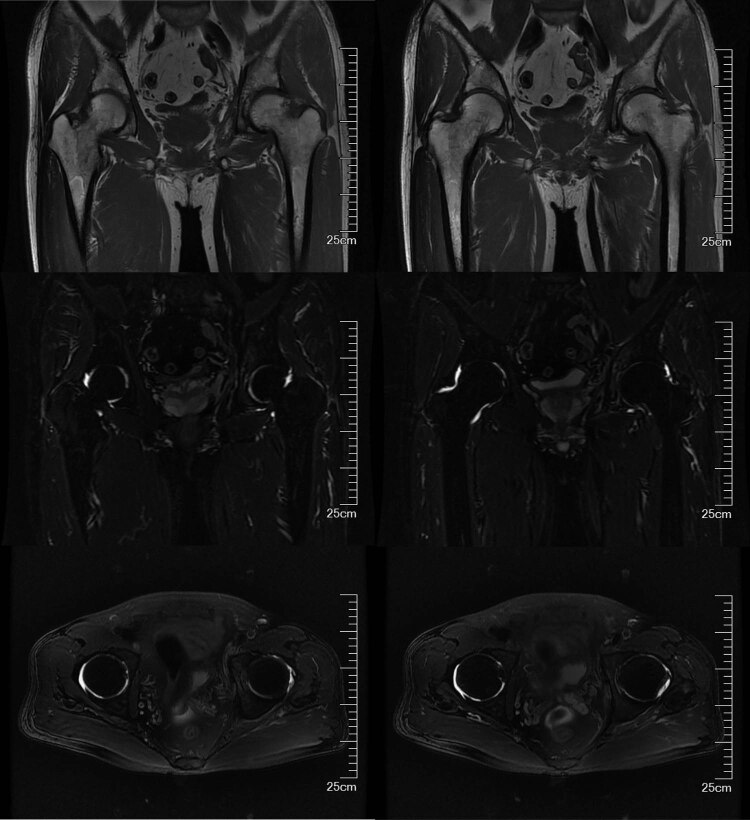
MRI of the hip joint shows a patchy hyperdense focus in the left acetabulum, a small amount of fluid signal shadow in the bilateral acetabulum, and no obvious swelling in the surrounding soft tissues.

**Figure 3:**
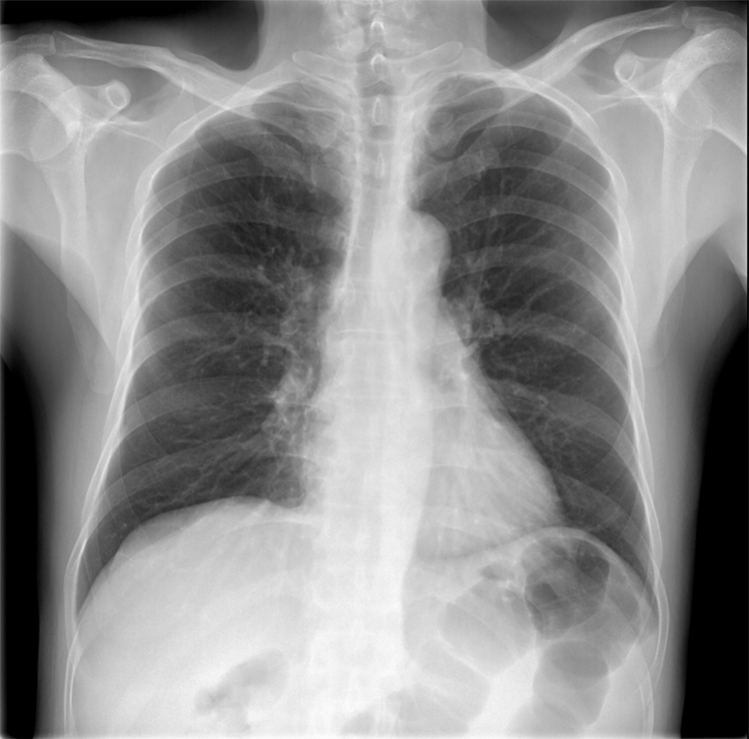
Chest radiographs show no obvious substantial lesions in both lungs.

**Figure 4:**
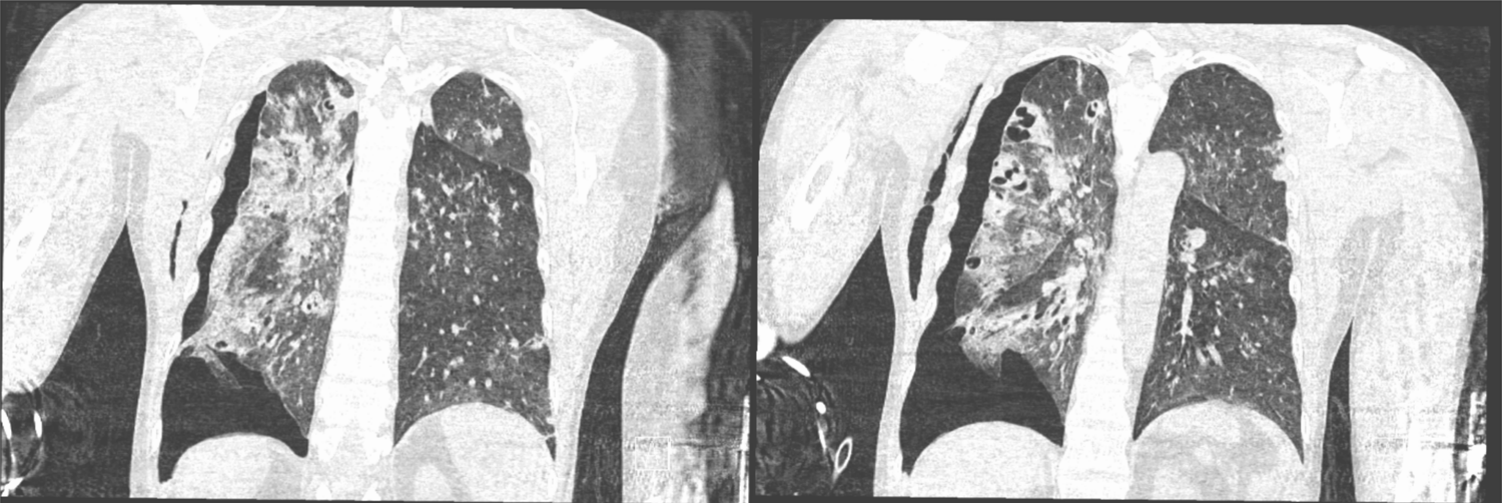
Lung computed tomography shows multiple pulmonary balloon lesions, and a *Staphylococcus aureus* infection was considered.

**Figure 5:**
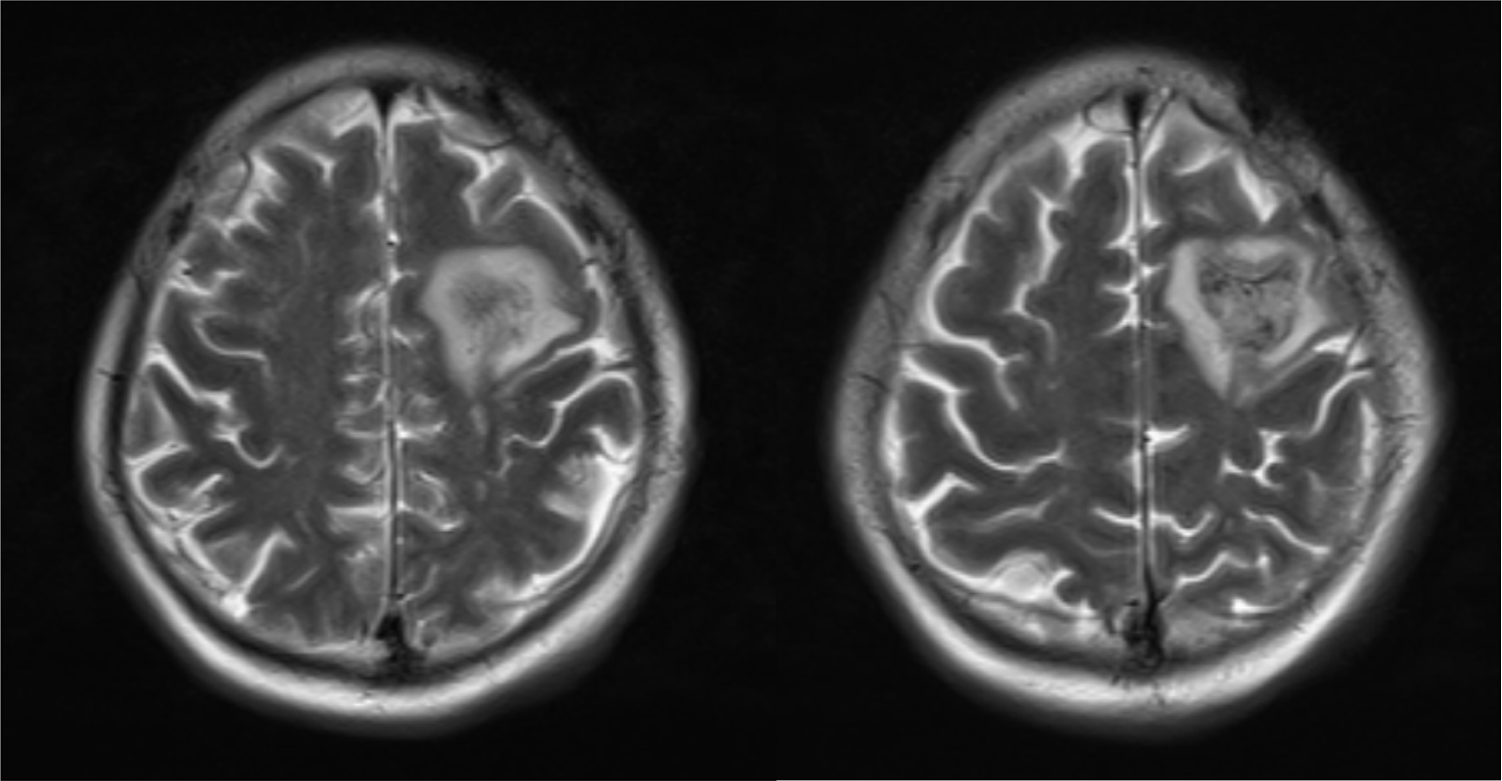
Head MRI shows abnormal signals in the left frontal lobe. Combined with the medical history, hemorrhagic infarction caused by bacterial embolism was considered. No obvious abscess cavity is seen, and the hemorrhage is in the subacute stage.

The results of auxiliary tests on the first day of admission were as follows: white blood cell (WBC) count, 9.22 × 10^9^/L (neutrophils, 67.9%; lymphocytes, 9.8%); hemoglobin concentration, 124 g/L; platelet count, 231 × 10^9^/L; erythrocyte sedimentation rate, 14 mm/h; and C-reactive protein, 10.2 mg/L. Pelvic plain radiographic images were suggestive of a deformed left femoral head, narrowed hip space, and osteophyte hyperplasia (Figure 1). Magnetic resonance imaging (MRI) revealed left acetabular bone marrow edema and a small amount of fluid in both hip joints (Figure 2). Chest radiograph showed no significant substantial lesions in both lungs (Figure 3). On admission, the patient was initially diagnosed with osteoarthritis of the left hip. Owing to the relatively obvious pain and left acetabular bone marrow edema, the patient’s condition was thought to be related to acetabular bone contusion caused by heavy physical labor. Joint replacement surgery was planned, with no administration of anti-infective therapy. On the 7th day after admission, the patient developed a persistently high retained fever over 38°C, and the hip joint pain was significantly aggravated. Laboratory tests revealed the following: WBC count, 13.42 × 10^9^/L (neutrophils, 8.38 × 10^9^/L); C-reactive protein, 381.1 mg/g; interleukin-6, >5000.0 pg/mL; and procalcitonin, 31.830 ng/mL. Left hip joint infection was accordingly suspected. After joint aspiration examination, blood culture, and drug sensitivity test, 1.5 g of cefuroxime sodium was given intravenously twice a day. After 5 consecutive days of treatment, this patient’s condition did not improve, and chest tightness, dyspnea, and decreased blood pressure occurred. The patient was transferred to the intensive care unit for mechanical ventilation, anti-infection, homeostasis, and organ function protection. Pulmonary computed tomography revealed multiple pneumonocytocystic changes in the lungs, suggestive of *S. aureus* infection (Figure 4), and joint puncture and blood culture confirmed the aforementioned suspicion. Considering the patient’s condition, we suspected *S. aureus* bacteremia had spread to various parts of his body, including the lungs (Figure 4) and brain (Figure 5). According to the results of the drug sensitivity test (Table 1), the antibiotic treatment regimen was adjusted to an intravenous drip of tigecycline at 100 mg for the first dose and 50 mg for each subsequent dose every 12 h, all while maintaining the stability of organ function, circulation, and acid-base balance. Nevertheless, continuous treatment for 2 weeks still had a poor therapeutic effect. Two weeks after admission, the patient developed multiple organ dysfunction in the respiratory, circulatory, nephrological, and pancreatic systems, as well as in other systems. His condition gradually deteriorated, and the patient died of multiple organ failure 1 month after admission.

**Table 1:** Results of blood culture and drug sensitivity test using the automated instrument method

Bacterial culture results∶ *Staphylococcus aureus*							
Antibiotic	Drug sensitivity	Results	Method	Antibiotic	Drug sensitivity	Results	Method
Teicoplanin	S	15	KB	Vancomycin	S	≤0.5	MIC
Microdata	S	≤16	MIC	Levofloxacin	S	0.25	MIC
Pediatric compound sulfamethoxazole tablets	S	≤10	MIC	Oxacillin sodium salt	S	0.5	MIC
Quinupristin/dalfopristin	S	≤0.25	MIC	Ciprofloxacin	S	≤0.5	MIC
Linezolid	S	2	MIC	RifaMpicin	S	≤0.5	MIC
Moxifloxacin	S	≤0.25	MIC	Clindamycin hydrochloride (Cleocin)	S	≤0.25	MIC
Gentamicin	S	≤0.5	MIC	Penicillin	R	≥0.5	MIC
Tigecycline	S	≤0.12	MIC	Tetracycline	S	≤1	MIC
Cefoxitin	—	Neg	MIC	Erythromycin	S	≤0.25	MIC

Note: Grade A sputum (WBC > 25/LP, epithelial cells < 10/LP) was qualified.

Type of resistance: 1; S, sensitive; R, resistant; I, intermediary. 2. MIC, minimum inhibitory concentration, indicating the lowest antibiotic concentration that can inhibit bacterial growth (μg/mL or mg/1); KB: Disk diffusion method. 3. Pos, positive; Ncg, negative.


**Informed consent:** Informed consent has been obtained from all individuals included in this study.
**Ethical approval:** The research related to human use has been complied with all the relevant national regulations, and institutional policies and in accordance with the tenets of the Helsinki Declaration, and has been approved by the Second Affiliated Hospital of Anhui Medical University.

## 3 Discussion

The annual incidence of infectious hip arthritis is reportedly less than 1 in 10,000 adults worldwide [5]. However, infectious hip arthritis remains a significant threat to human health [6]. Through a retrospective cohort study on the mortality and reinfection rate of patients with septic hip arthritis postoperatively, Kao et al. found that the mortality and recurrent infection rate at 2 years were higher after surgical treatment of primary septic hip arthritis compared to other types of surgery in adults [7]. Balato et al. have shown that hip septic arthritis can cause pain and disability in patients, with a mortality rate of approximately 10%. Methicillin-sensitive *S. aureus* is the most common pathogen of hip septic arthritis [8]. Huang et al. found through a cohort study that, among the strains of septic arthritis, the incidence of septic arthritis of the hip was second only to that of septic arthritis of the knee (14.4%), with the first predicted cause of death of septic arthritis being hip infection [9]. The diagnosis of typical suppurative arthritis is relatively simple in a typical patient with fever, stiffness, increased joint skin temperature, swelling, and severe pain. However, the clinical and laboratory presentation of infectious arthritis is often atypical, with only 58% of patients having high-grade fever and only 50–60% having serum leukocytosis [10]. Approximately 50% of cases of suppurative arthritis are caused by *S. aureus*. The most common route of infection is transmission via the blood. Additionally, bacteria can enter the joints directly via punctures, surgery, and injury or indirectly through infections in adjacent tissues.

*S. aureus* is an invasive pathogen. Following the joint invasion, it rapidly proliferates, triggering an acute inflammatory response, and produces several virulence factors that promote its spread, causing acute suppurative arthritis [11,12]. Although the clinical symptoms of *S. aureus* are similar to those of most other suppurative arthritis types, they have a more rapid onset, and patients usually experience chills and fevers up to 40°C. The patient’s blood leukocyte and neutrophil counts were elevated. There was joint redness, swelling, warmth, tenderness, abscesses in the joint capsule, and cellulitis or abscess formation in the surrounding tissues. A diagnosis of septic arthritis was confirmed by smear and culture of the aspirated fluid from the joint cavity. The radiographic features of these infections are unspecific early on, yet MRI generally reveals lesions earlier than radiography. Previously, infectious lesions of the hip were rarely considered as a differential diagnosis of hip pain since degenerative changes are a more common cause of hip pain. A poor understanding of inflammatory hip disease leads to delayed diagnosis, which may lead to irreversible pathological changes in the hip. According to the revised Newman criteria, infectious hip arthritis is usually diagnosed in the presence of one of these four conditions: (1) isolation of the pathogenic organism from the infected joint; (2) isolation of pathogenic microorganisms from other sources (such as blood), as well as symptoms of local joint inflammation such as redness, swelling, heat, and pain; (3) typical clinical features and a past history of antibiotic treatment due to articular fluid opacity; and (4) necropsy or pathologic features suggestive of suppurative arthritis [13]. In the current study, the patient did not show the typical clinical signs, symptoms, and imaging features that are characteristic of the early stage of the disease; therefore, we were unable to make a timely and accurate diagnosis. Because the disease progressed rapidly, the opportunity for optimal treatment was missed, causing mortality. Early diagnosis and treatment are extremely important for patients with *S. aureus* arthritis because the disease progresses more rapidly than other infectious arthritis. Delayed treatment can lead to joint degeneration, osteonecrosis, and joint instability, which could lead to systemic sepsis and death through hematogenous transmission [6].

The ideal treatment for suppurative hip arthritis remains controversial; however, early accurate diagnosis and timely treatment are keys to successful disease management because of the rapid destruction of the hip and hematogenous metastasis. Treatment options that have been proven to be effective include arthroscopy, open debridement, and a two-stage strategy; stage one involves the resection of the arthroplasty and implantation of an antibiotic cement spacer, and stage two involves total hip replacement [14,15,16,17,18]. Each treatment has significant advantages and disadvantages. The criteria for the successful treatment of bacterial arthritis are the eradication of infection, absence of reinfection or infectious complications, and recovery of joint function. Therefore, antibiotic therapy is the necessary basic treatment, and antibiotics should be selected rationally according to culture and drug sensitivity results. However, until test results are obtained, empirical antibiotic treatment should be administered [19]. Early and adequate antibiotic treatment is protective, but late treatment is not and may even exacerbate immune system damage, leading to uncontrolled infection and death [20].

In conclusion, the treatment of suppurative arthritis of the hip joint should be based on the findings of the given case. However, due to atypical cases, as in our case, early hip pain may not receive enough attention, avoiding joint puncture examination and blood culture. Since we did not get an accurate timely diagnosis, the patient did not receive timely and effective treatment, including antibiotic treatment for the source of infection, and died. Therefore, early diagnosis and timely treatment are key to a good prognosis.
